# The Educational Treatment of Idiots, Imbeciles, and Feeble Minded Children

**Published:** 1894-09-08

**Authors:** G. E. Shuttleworth

**Affiliations:** late Medical Superintendent, Royal Albert Asylum, Lancaster


### The Educational Treatment of Idiots, Imbeciles, and Feeble-Minded Children.

By G. E. Shuttleworth, B.A., M.D., &c., late Medical Superintendent, Royal Albert Asylum, Lancaster.

### V.—RESULTS OF SPECIAL TRAINING.

In this, our final paper on the above subject, we propose to sum up, so far as information gathered from some considerable practical experience, as well as from divers Reports, will serve, the results attained by the special training we have endeavoured to describe. Some of our readers there are no doubt who are already inclined dubiously to inquire "*Cui bono?*" Others again will perhaps anticipate, as parents of defective children usually do, greater results from the elaborate system of training than are really practicable. It is, in truth, impossible to make a "silk purse out of a sow's ear," yet a passable receptacle for at least small coin may be made by careful manipulation of the latter. So with the imbecile; whilst the instructor cannot reasonably be expected to put into the mental capacity of the pupil that which (as a parent once quaintly expressed it) "Providence has left out," he can, if he knows how, develop such faculties as do exist, though in a very rudimentary condition. In this process the medico-psychologist and the teacher must go hand in hand; the former points out the defects and indications for training which the latter carries into practical effect. In some instances (as we have seen) remarkable ability in certain directions is found, associated, however, with a lack of mental balance, or defect of control, which renders the special gift comparatively useless. In such cases attention must be given not only to the special gift but to the development of the other mental powers requisite for its orderly employment. Thus a youth endowed with a marvellous mechanical memory for numbers, at first expended somewhat uselessly in the recollection of the birthdays—days of the week as well as dates for years past—of all with whom he came into contact, has been trained to be a trustworthy assistant to the storekeeper of the institution where he was brought up. Another whose taste for form and colour was displayed in the arrangement of a play-box of coloured tiles, has by systematic instruction been qualified for the post of designer in a Lancashire mill. Where, however, no particular talent can be found, there is, as Lord Herschell well remarks, usually "some spark of intelligence which might be fanned into a flame. Difficult enough it might be to discover the particular spot where that spark rested, but experience, and a loving care and watchfulness, triumphed over these difficulties. Those who in times gone by would have been regarded as doomed to a life of hopeless imbecility, have been now rendered in some cases as capable of thinking, reasoning, and taking part in the battle of life as their fellow-men, while in a great number of cases, though defects existed, the patient had been so much improved as to be a useful and happy member of society." This weighty expression of opinion by the Lord Chancellor (delivered at the Royal Albert Asylum festival in 1888) agrees in a striking manner with Séguin's declaration after thirty years' practical experience. His testimony is that "idiots have been improved, educated, and even cured; not one in a thousand has been entirely refractory to treatment; not one in a hundred who has not been made more happy and healthy; more than 30 per cent. have been taught to conform to social and moral law, and rendered capable of order, of good feeling, and of working like the third of a man; more than 40 per cent. have become capable of the ordinary transactions of life under friendly control, of understanding moral and social abstractions, of working like two-thirds of a man; and 25 to 30 per cent. come nearer and nearer the standard of manhood, till some of them will defy the scrutiny of good judges when compared with ordinary young men and women." That this is no mere rhetorical flourish is proved by the statistics of one of the largest English training institutions for imbeciles. Systematic inquiry having been made from year to year as to the after career of pupils discharged on completion of their seven years' course of training, it was found that 10 per cent. were, or had been, earning wages; 5 per cent. were remuneratively employed at home; and 3·5 per cent. were (in their friends' opinion) capable of earning wages if suitable situations could be found for them. About 22 per cent. were reported to be more or less useful to their friends at home, while another 22 per cent. were reported as of little or no use; 29 per cent. had gravitated to workhouses and lunatic asylums; the remainder (8·5 per cent.) had died. There is no doubt that much useful training is wasted when, as in the homes of the poor, there is no longer opportunity for the improved imbecile practising what has been laboriously taught him; and the institution of industrial homes for such, as well as for the class of "feeble-minded" above the standard of institutions for idiots, is a great desideratum. Happily, an encouraging start has been made in this direction by the establishment of several small homes for feeble-minded girls, where the cost of maintenance is partly defrayed by the proceeds of their laundry-work. The special need of protection for weak-minded females to prevent the evil being transmitted to another generation is obvious; but it is hoped that similar provision may before long also be made for what nowadays we may call the "inferior sex!"

Had our space permitted, it would have been interesting to refer to the efforts made of late years for the benefit of those mentally-feeble, as distinguished from the category of idiot and imbecile. Some account has already appeared in The Hospital (April 28th, 1890, p. 70) of the investigation undertaken by Dr. Francis Warner and his colleagues into the physical and mental condition of 100,000 school-children, the gross result of which may be stated as showing that special instruction is requisite for about 1·6 per cent. of the number. It is a matter of satisfaction that a good example in this direction has been set by the School Board for London, and that at the present time upwards of 500 mentally abnormal children are being specially instructed on principles similar to those described in our earlier articles, in some dozen centres in different parts of the metropolis. Of these, as well as of the duller children in institutions, we may well observe, in the language of Prospero, that

> "As the morning steals upon the night,
> Melting the darkness, so their rising senses
> Begin to chase the ignorant fumes that mantle
> Their clearer reason."

